# Delineation of molecular characteristics in pediatric PFA ependymoma involving rare osseous and pulmonary metastases: A case report and literature review

**DOI:** 10.3389/fonc.2022.1001118

**Published:** 2022-11-15

**Authors:** Mading Zhou, Leiming Wang, Peng Sun, Yutong Liu, Ge Chen, Gao Zeng

**Affiliations:** ^1^ Department of Neurosurgery, Xuanwu Hospital, Capital Medical University, Beijing, China; ^2^ Department of Pathology, Xuanwu Hospital, Capital Medical University, Beijing, China

**Keywords:** PFA, pediatric ependymoma, extraneural metastases, CDK12, chemotherapy

## Abstract

Ependymoma is the third most common pediatric primary brain tumor, with its most aggressive subtype being posterior fossa group A (PFA). Extraneural metastasis of pediatric PFA ependymoma is rare. Herein, we present a case of a 9-year-old girl with PFA ependymoma characterized by a lack of trimethylation of histone H3 at lysine 27 and elevated chromosome X open reading frame 67 expression. Despite multiple surgeries and radiotherapies, the patient had a rapid recurrence and developed osseous and pulmonary metastases, which may be attributed to the homozygous deletion of cyclin-dependent kinase (CDK) inhibitor 2A/B and CDK12 mutation. Importantly, the CDK12 mutation observed in the patient may be indicative of the need for further work-up to consider chemotherapy rather than administering poly (adenosine diphosphate-ribose) polymerase inhibitors. Taken together, this is the first report of pediatric PFA ependymoma with extraneural metastases, wherein we clarified the diagnostic procedures of this newly identified PFA ependymoma and provided new cues to study the invasiveness of this disease and treatment selection for such patients.

## Introduction

Ependymoma is the third most common pediatric primary brain tumor, and 90% of childhood ependymoma occurs intracranially, with one-third occurring in the supratentorial area and two-thirds occurring in the posterior fossa (PF) (1). The 2021 edition of the World Health Organization (WHO) classification of central nervous system (CNS) tumors refined approaches to classifying ependymoma based on gene expression profiles and deoxyribonucleic acid methylation status due to an increasing understanding of the genomic landscape of tumors of the CNS (2). PF ependymoma is classified into three subtypes, namely PF group A (PFA), PF group B, and PF subependymoma, each having different demographics, epigenetics, transcriptomes, and outcomes. Among these, PFA ependymoma, characterized by lack of trimethylation of histone H3 at lysine 27 (H3 K27-me3) and elevated chromosome X open reading frame 67 (CXorf67) expression, is the commonest and aggressive form (2, 3). Previous studies revealed that PFA ependymoma comprises ependymal cells with poor differentiation and active mitosis; therefore, the tumors have frequent local relapses and might metastasize to distant brain and spinal canal areas through the cerebrospinal fluid (1). However, extraneural metastasis of pediatric ependymoma is rare (4–6).

Herein, we report the case of a 9-year-old girl with PFA ependymoma characterized by lack of H3 K27-me3 and elevated CXorf67 expression. Despite undergoing multiple tumor resections and radiotherapies, the patient had a rapid recurrence and developed osseous and pulmonary metastases. Besides resecting the metastasis lesion in the sphenoid sinus by transnasal endoscopic surgery, we gathered tumor specimens to perform genomic analysis. Genomic analysis revealed that the patient harbored cyclin-dependent kinase (CDK) 12 mutation and homozygous deletion of CDK inhibitor 2A/B (CDKN2A/B). We also performed a systematic review of the literature to identify potential factors leading to extraneural metastasis of PFA ependymoma.

## Case description

**Figure 1:**
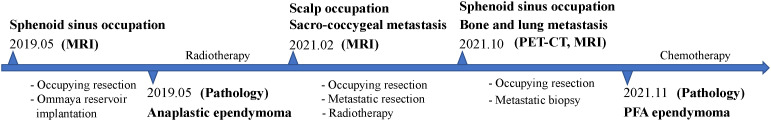
Timeline of disease diagnosis and treatment.

A 9-year-old girl visited our department in October 2021 owing to bilateral abducent paralysis. The timeline of the disease diagnosis and treatment are presented in **Figure 1**.

The patient first visited a local hospital on May 2019 owing to headache, nausea, and vomiting, and magnetic resonance imaging (MRI) revealed a tumor in the PF (**Figures S1A-C**). Ommaya reservoir implantation and subtotal resection of the tumor was performed at the local hospital (**Figures S1D-F**), and an initial pathological diagnosis of anaplastic ependymoma (WHO grade III) was established. The patient subsequently received adjuvant radiotherapy (50.4 Gy at 1.8 Gy per fraction over 4 weeks) in Japan. Unfortunately, an MRI performed in February 2021 revealed scalp and sacrococcygeal metastases (**Figures S2A-D**). Later in March 2021, the patient underwent metastases (scalp and sacrococcygeal) resection at the local hospital, followed by whole-spine radiotherapy (**Figures S2E-H**). When the patient had bilateral abducent paralysis in October 2021, she underwent whole-body positron emission tomography/computed tomography and brain MRI at our hospital. The results revealed sphenoid sinus, osseous, and pulmonary metastases (**Figures S3A-D**). The osseous metastasis was then confirmed by right femur biopsy, followed by histopathologic and immunohistochemical analysis (**Figure S4** **).** Furthermore, transnasal endoscopic surgery was performed to resect the sphenoid sinus metastasis (**Figures S3E-G**), and the specimen was sent for pathological examination and genomic analysis.

**Figure 2:**
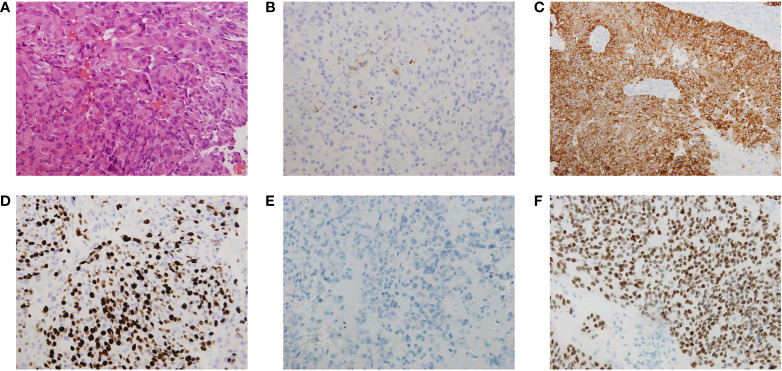
Histological and immunohistochemical analysis of the sphenoid sinus lesions. **(A)** Sheets of monotonous cells presenting as uniform, bland nuclei with evenly distributed chromatin, perivascular pseudorosettes and necrotic zones were observed on hematoxylin and eosin staining analysis. The tumor cells showed **(B)** dot-like epithelial membrane antigen (EMA) immunoexpression and **(C)** diffuse cytoplasmic glial fibrillary acidic protein (GFAP) immunoexpression suggestive of ependymal differentiation. **(D)** Ki-67 labelling index in the sphenoid sinus tumor was 60%, which showed a high proliferation fraction of the tumor cells. The tumor cells showed **(E)** immunonegative H3 K27-me3 staining and **(F)** immunopositive CXorf67 staining. Vascular endothelial cells in the tumor section were used as the positive control for H3 K27-me3 staining.

Histological examination revealed a tumor measuring 4×3×1.5 cm, with sheets of monotonous cells presenting as uniform, bland nuclei with evenly distributed chromatin on hematoxylin and eosin staining (**Figure 2A**). Perivascular pseudorosettes and necrotic zones were observed. The tumor cells showed dot-like epithelial membrane antigen immunoexpression (**Figure 2B** **)** and diffuse cytoplasmic glial fibrillary acidic protein immunoexpression (**Figure 2C** **)** suggestive of ependymal differentiation. Ki-67 showed a high proliferation fraction of the tumor cells (**Figure 2D**). Immunohistochemical staining was negative for H3 K27-me3 staining (**Figure 2E**) and positive for CXorf67 staining (**Figure 2F**). A final diagnosis of PFA ependymoma (WHO grade III) was established based on the histopathologic and immunohistochemical features, particularly the characteristic lack of H3 K27-me3 and elevated CXorf67 expression.

The tumor specimen removed from metastatic lesions of the sphenoid sinus was analyzed using next-generation sequencing to identify potential therapeutic targets. DNA extracted from plasma was used as methodological reference for eliminating the interference from germline mutation. The 833 genes based Onco PanScan™ panel identified CDK12: p.Gly1271AspfsTer (allelic frequency = 0.012) as a loss of function mutation. A homozygous deletion spanning the CDKN2A/B locus at 9p21 was also identified in the tumor sample using multicolor interphase fluorescence *in situ* hybridization analysis (**Figure S5**). The patient was then referred to a local oncology center, where she received conventional chemotherapy (etoposide + ifosfamide + cisplatin for five cycles and etoposide + ifosfamide + nedaplatin for seven cycles). After 12 cycles of chemotherapy, the patient had stable disease (SD).

## Discussion

Ependymoma, the third most common pediatric primary brain tumor, has a poor prognosis. Up to 50% of patients with ependymoma experience a relapse at the primary site, occasionally accompanied by metastasis within the CNS. However, based on our systematic literature review, pediatric ependymoma rarely metastasizes extraneurally, and to date, only 13 cases have been reported (including the present case) (5, 7–17) (**Table S1**). Of these, our report is the first and only case report of a PFA ependymoma with extraneural metastases. A lack of this finding might result in unnecessary diagnostic considerations, resulting in patients missing treatment opportunities. Herein, we reported a case that relapsed rapidly, with widespread osseous and pulmonary metastases from a PFA ependymoma with homozygous deletion of CDKN2A/B and CDK12 mutation.

**Figure 3:**
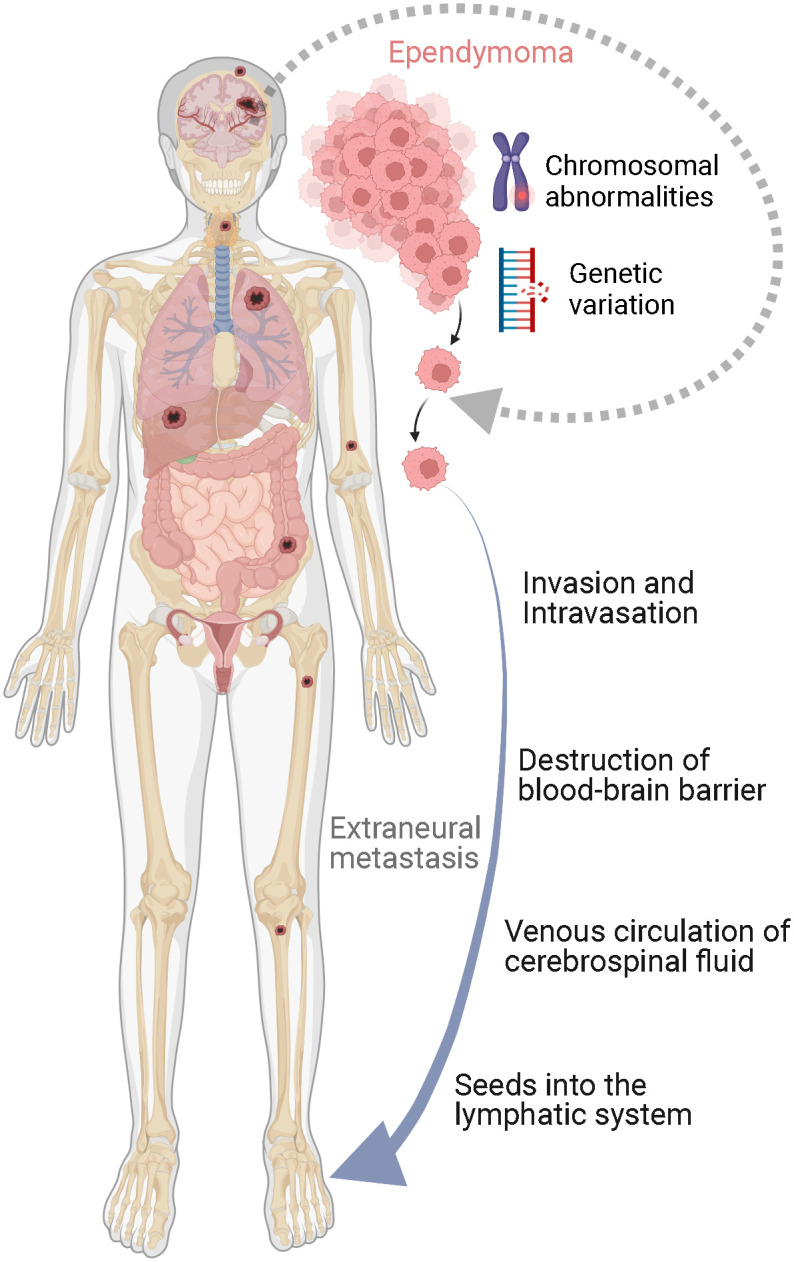
Illustration of mechanisms for extraneural metastasis of ependymoma.

Although several theories exist on the mechanism for extraneural spread, the exact mechanism by which ependymoma metastasizes extraneurally remains unknown. By performing a systematic review of the literature, we summarized the potential mechanisms for extraneural metastasis of ependymoma (**Figure 3**). Except for the molecular characteristics of tumor cells, including chromosomal abnormalities and genetic variations, four valuable potential extracellular mechanisms exist, which are as follows: (1) dissemination caused by tumor cells penetrating the scalp from the surgical site into the vascular or lymphatic system; (2) dissemination of tumor cells caused by the destruction of the blood-brain barrier following craniotomy and shunt surgery; (3) dissemination of tumor cells *via* the spread from the arachnoid villi of the CNS to the superior sagittal sinus and systemic venous circulation; (4) dissemination of tumor cells caused by the extension of the tumor to the skull structure and seeding into the lymphatic system (5, 16). Nevertheless, no study has identified the exact mechanism by which PFA ependymoma metastasizes. A recent study of a 7-year-old boy diagnosed with PF ependymoma reported lung metastases after surgical resection and ventriculoperitoneal shunt insertion and proposed that the lung metastasis might have been caused by tumor invasion into the dural venous sinus or diffusion after atrioventricular shunt implantation (5). In addition, recent studies reported that high-risk PFA ependymoma with poor outcomes, such as multiple recurrences, tended to have some genetic characteristics, including the gain of chromosome 1q, loss of chromosome 6q, and CDKN2A/B deletion (18–22). Among them, CDKN2A/B is the second most frequently inactivated tumor suppressor gene in human cancers, the inactivation of which reportedly affects tumor growth and metastasis.

In the current case, we speculated that osseous and pulmonary metastases might be attributed to the dissemination caused by tumor cells penetrating the scalp from the surgical site into the vascular or lymphatic system, considering that the patient had scalp occupation less than 2 years after the surgery. This is because the retrosigmoid approach for PF tumor resection was adopted during the first surgery at the local hospital. Furthermore, the high metastatic capacity of the tumor cells in the patient might theoretically be attributed to the homozygous deletion of CDKN2A/B and CDK12 mutation; however, no characteristic chromosomal abnormalities were observed. CDK12, as a member of the CDK family of serine/threonine protein kinases, regulates transcription and post-transcriptional processes, thereby regulating various cellular functions. Genomic alterations in CDK12 have been detected in breast, stomach, esophageal, and other cancers, with the characteristics of oncogenesis (23, 24). In addition, recent studies have demonstrated the aggressive behavior of CDK12 mutation. Melissa et al. reported that prostate cancer patients with CDK12 mutation undergo metastasis in a shorter time (25). Zhang et al. reported that CDK12 mutation is associated with osseous metastasis of non-small cell lung cancer (26). Therefore, we speculated that in addition to CDKN2A/B deletion, CDK12 mutation was associated with metastasis in pediatric PFA ependymoma.

Molecular profiling is gaining popularity in pediatric CNS tumors for supporting diagnosis, prognosis, and therapeutic decisions. Given that PFA ependymoma is known as epigenetically deregulated tumors, several studies proposed that epigenetic-related drugs, such as poly (adenosine diphosphate-ribose) polymerase (PARP) inhibitors, might serve as promising therapeutic drugs (3, 27). Our patient also met the inclusion criteria of a clinical trial on a PARP inhibitor (Nilaparil, ChiCTR2100051395) in China. However, due to the CDK12 mutation in our patient, the efficacy of PARP inhibitors is unknown. Although a preclinical study demonstrated that CDK12 deficiency might increase tumor cell sensitivity to PARP inhibitors in breast cancer gene (BRCA) 1- or BRCA2-mutant tumors *via* genetic synthetic lethality (28), recent studies revealed CDK12 mutation might correlate with inferior response to PARP inhibitors and increased sensitivity to platinum-based chemotherapy (29, 30). In addition, our patient and her family refused targeted therapy and received conventional chemotherapy owing to their reluctance to clinical trials. After 12 cycles of chemotherapy, the patient had SD.

## Conclusion

To the best of our knowledge, this is the first study to report the case of a pediatric PFA ependymoma with extraneural metastases (osseous and pulmonary metastases). We further clarified the diagnostic procedures of this newly identified PFA ependymoma and provided new cues for the study of the disease invasiveness and treatment selection for such patients.

## Data availability statement

The raw data supporting the conclusions of this article will be made available by the authors.

## Ethics statement

The human tissue study protocol was approved by the Ethics Committee of Xuanwu Hospital, Capital Medical University. Written informed consent to participate in this study was provided by the participants’ legal guardian/next of kin. Written informed consent was obtained from the minor(s)’ legal guardian/next of kin for the publication of any potentially identifiable images or data included in this article.

## Author contributions

MZ: drafting/revision of the manuscript for content including medical writing for content. LW: interpretation of the pathological finding. PS: major role in the acquisition of data and performed the endoscopic surgery. YL: major role in the acquisition of data. GC: performed the endoscopic surgery and major role in the acquisition of data. GZ: study concept or design and Analysis or interpretation of data. All authors contributed to the article and approved the submitted version.

## Acknowledgments

We are grateful to the patient and the families of the patient who have made this research possible. We thank Bullet Edits Limited for language editing of the manuscript. We also thank Genetron Health Inc., that supported genomic studies.

## Conflict of interest

The authors declare that the research was conducted in the absence of any commercial or financial relationships that could be construed as a potential conflict of interest.

## Publisher’s note

All claims expressed in this article are solely those of the authors and do not necessarily represent those of their affiliated organizations, or those of the publisher, the editors and the reviewers. Any product that may be evaluated in this article, or claim that may be made by its manufacturer, is not guaranteed or endorsed by the publisher.

## Supplementary material

Supplementary Table 1 Summary of pediatric ependymoma cases with extraneural metastasis.
