# Supplementary material for: Delineation of molecular characteristics in pediatric PFA ependymoma involving rare osseous and pulmonary metastases: A case report and literature review
Source: Front Oncol. 2022 Nov 15;12:1001118. doi: 10.3389/fonc.2022.1001118 (PMC9706190; doi:10.3389/fonc.2022.1001118)
Supplement: Supplementary file 1 [file DataSheet_1.docx]

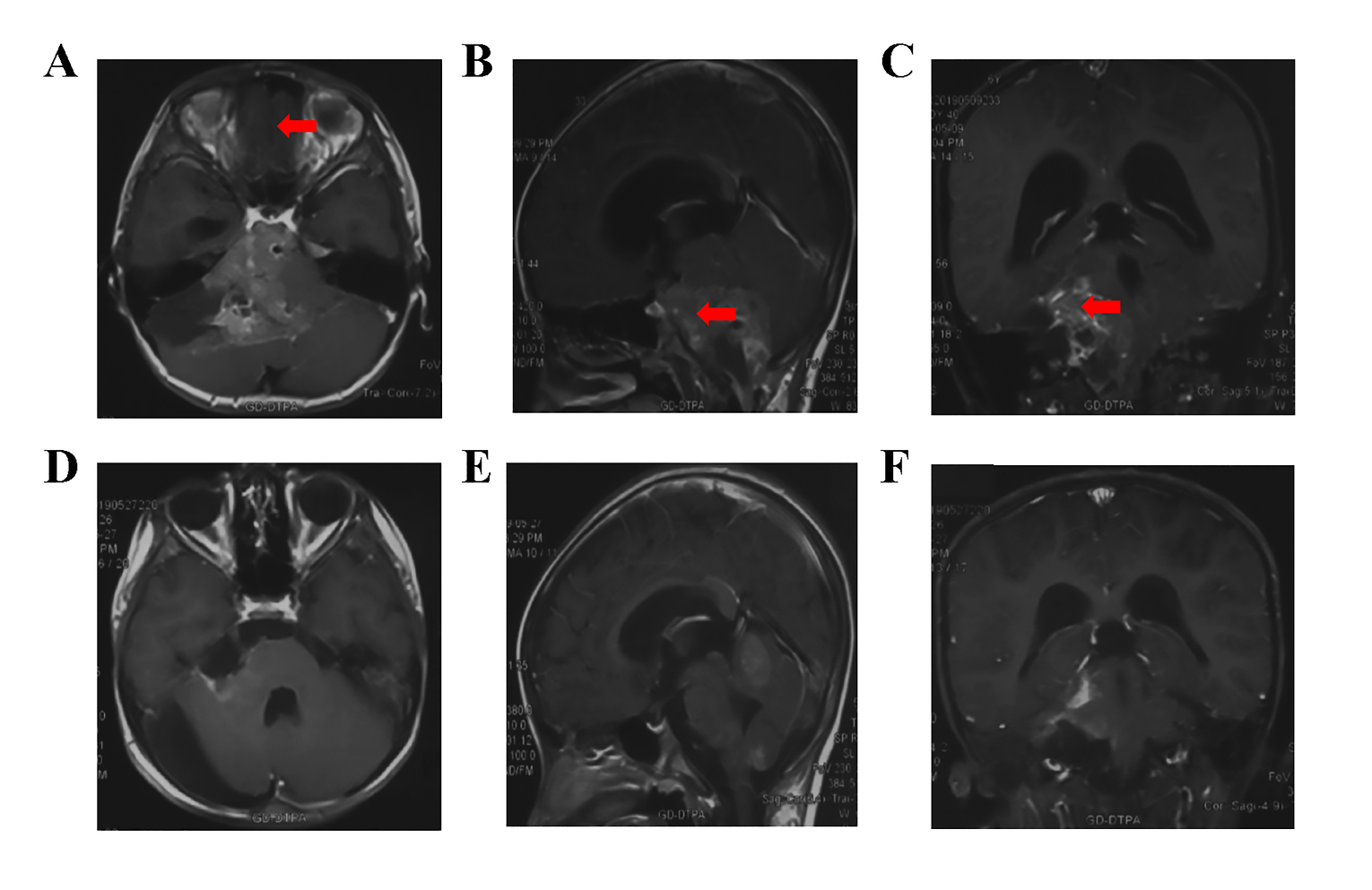


**Figure S1. MRI images of PF anaplastic ependymoma before and after surgery.** (A) axial, (B) sagittal, and (C) coronal images of sphenoid sinus lesions (red arrows) before surgery. (D) axial, (E) sagittal, and (F) coronal images of sphenoid sinus lesions after surgery.


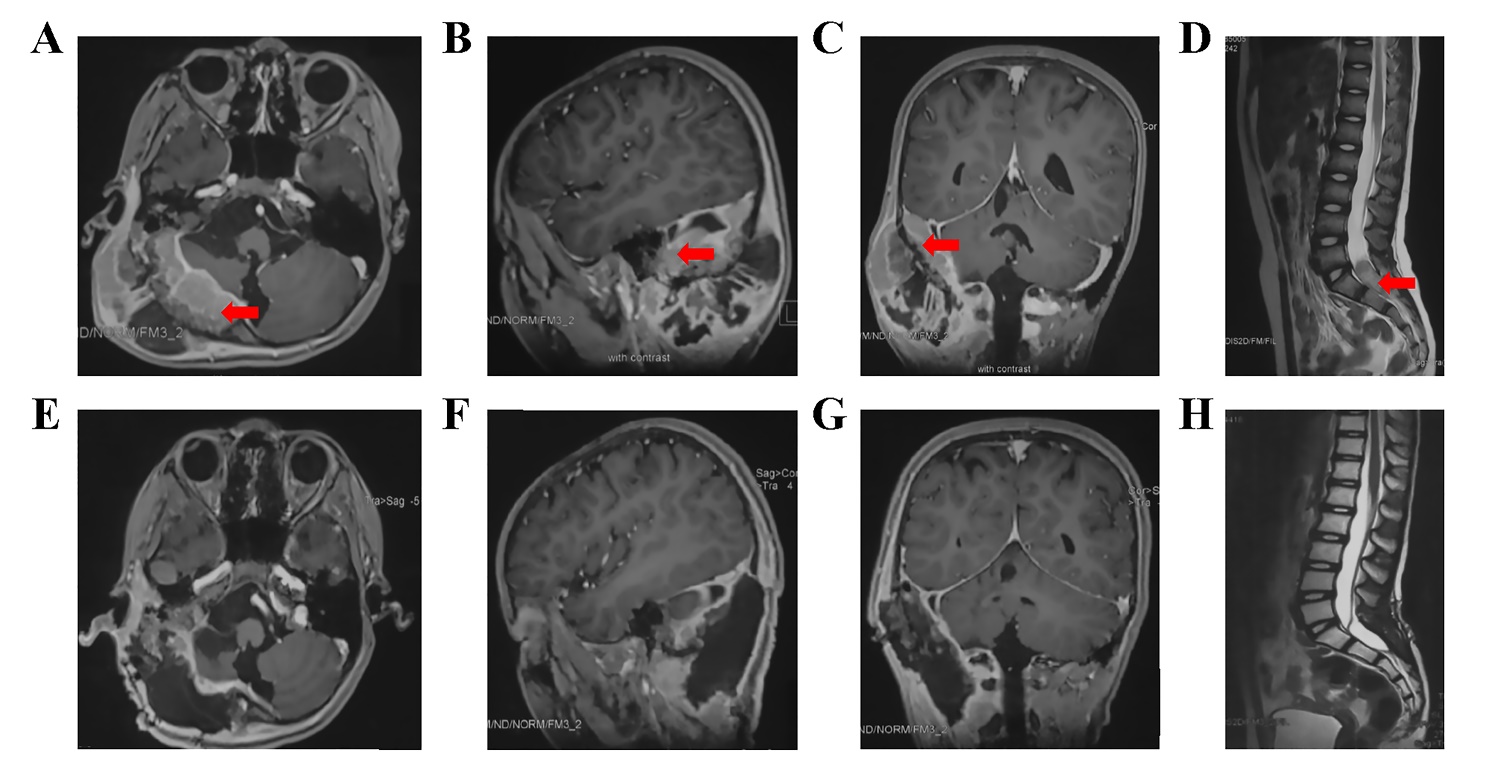


**Figure S2.** **MRI images of scalp occupation and sacro-coccygeal metastasis before and after surgery.** (A) axial, (B) sagittal, and (C) coronal images of scalp occupation lesions (red arrows) before surgery. (E) axial, (F) sagittal, and (G) coronal images of scalp occupation lesions after surgery. (D and H) MRI images of sacro-coccygeal metastasis lesions (red arrows) before (D)and after surgery (H).


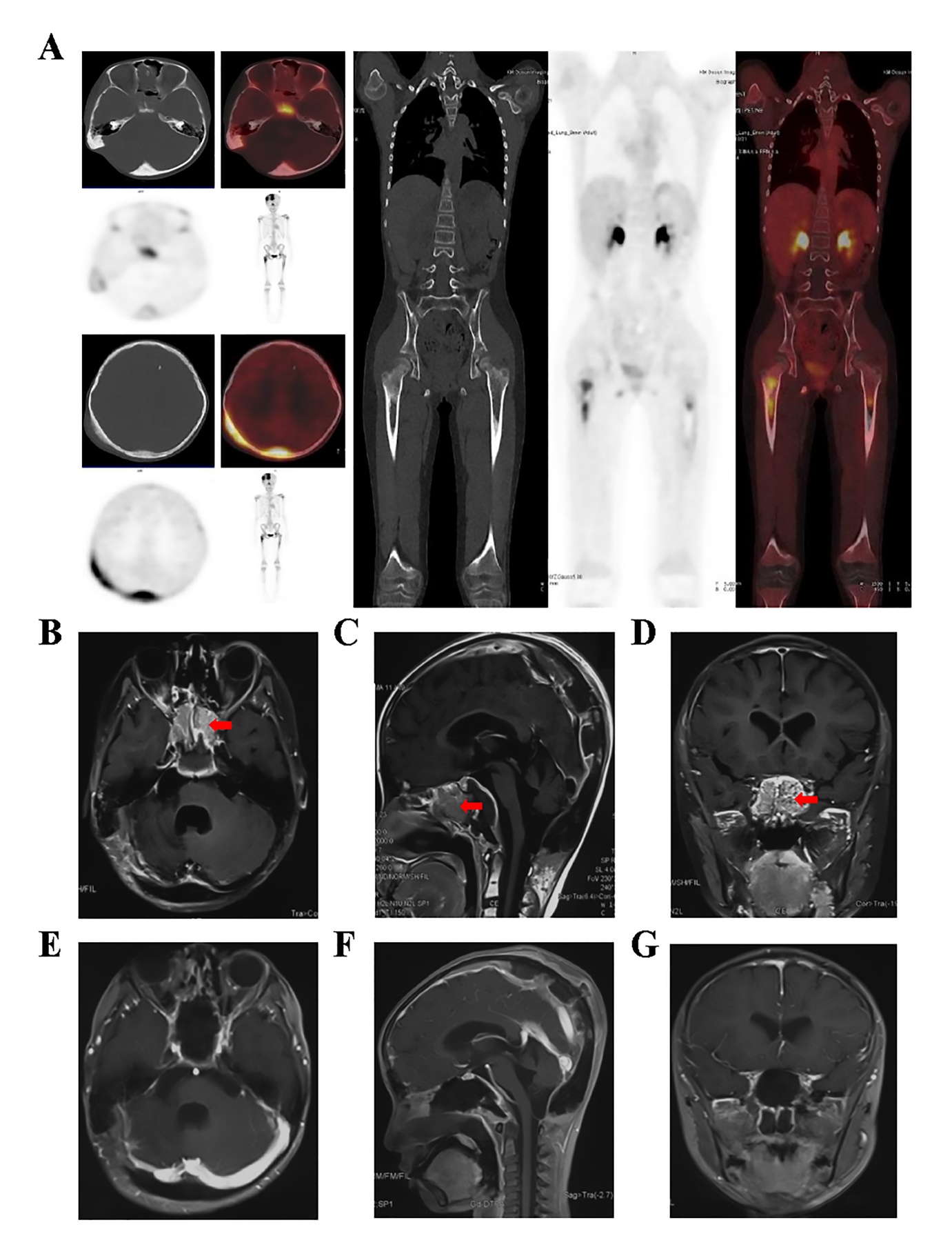


**Figure S3. Images of sphenoid sinus relapse and distant metastasis lesions.** (A) PET-CT images of multiple metastases in the whole body. (B) axial, (C) sagittal, and (D) coronal images of sphenoid sinus lesions (red arrows) before surgery. (E) axial, (F) sagittal, and (G) coronal images of sphenoid sinus lesions after surgery.


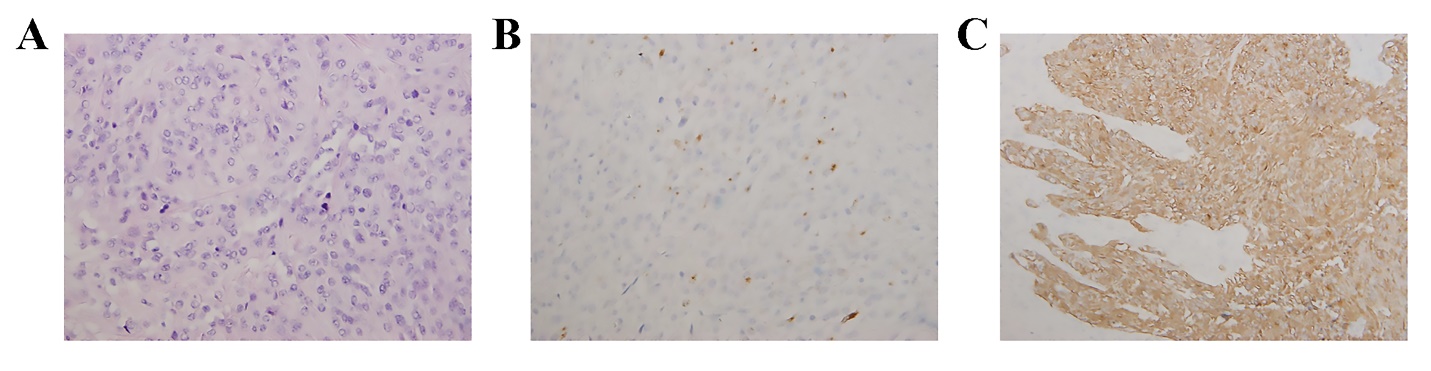


**Figure S4. Histological and immunohistochemical analysis of the bone metastasis.** (A) Biopsy showed islands of tumor cells separated by new born bone trabecula. The tumor cells showed (B) dot-like EMA immunoexpression and (C) diffuse cytoplasmic GFAP immunoexpression suggestive of ependymal differentiation.

**
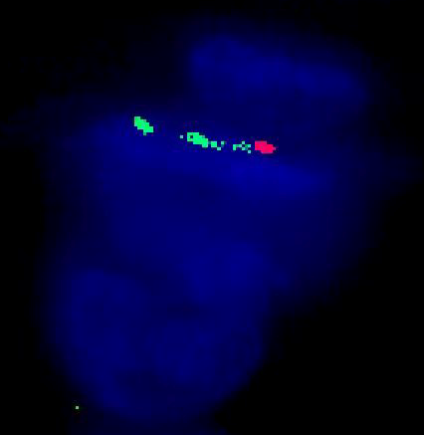
**

**Figure S5. Image of cyclin-dependent kinase inhibitor 2A/B (CDKN2A/B) gene in the multicolor interphase fluorescence in situ hybridization analysis.** The green signal (G) represents centromeric probe (CSP 9), and the red signal (R) represents site-specific probe (GSP CDKN2A/B). The signal mode in this figure is 2G1R, indicating the homozygous deletion of CDKN2A/B.

**Table S1. Summary of pediatric ependymoma cases with extraneural metastasis.**

| Study ID | Age  (years) | Gender | Location of Primary tumor | Treatment of Primary tumor | Intracranial Recurrence | Sites of extraneural metastasis | Treatment of extraneural metastasis | Survival time (years) |
| --- | --- | --- | --- | --- | --- | --- | --- | --- |
| Breslich et al. (1957) | 13 | Male | Right occipital lobe | Surgery+ Radiotherapy | NA | Right lung, pleura, hilar nodes | NA | 2 |
| Newton et al. (1992) | 3 | Male | Right thalamus into third ventricle | Radiotherapy+ Chemotherapy | Yes | Thoracic lymph nodes, peritoneum, | Resection+ Chemotherapy | >3 |
|  | 3 | Male | Right cerebrum | Surgery | Yes | Lung, mediastinum, thoracic lymph nodes, pleural space | None | 1.5 |
| Kinoshita et al. (2004) | 11 | Female | Right frontal lobe | Surgery+ Radiotherapy | Yes | Cervical lymph nodes | Resection+ Chemotherapy | >5 |
| Varan et al. (2006) | 8 | Male | Distal spinal  cord | Surgery+ Radiotherapy | NA | Liver | Chemotherapy | 1.35 |
| Kumar et al. (2007) | 10 | Male | Right occipital horn of lateral ventricle | Surgery | Yes | Right scalp, right cervical lymph nodes | Resection+ Chemotherapy | >10 |
| Hussain et al. (2010) | 6 | Female | Left frontal lobe | Surgery + Radiotherapy | Yes | Bone | Chemotherapy | 6 |
| Chao et al. (2011) | 10 | Male | Left parietal lobe | Surgery | Yes | Scalp, cervical lymph nodes | Chemotherapy | 4.5 |
| Fischer et al. (2013) | 6 | Female | Left parietal lobe | Surgery + Radiotherapy | Yes | Bone, lung, liver, lymph nodes | Radiotherapy+ Chemotherapy | 3 |
| Alzahrani et al. (2014) | 7 | Male | Posterior fossa | Surgery + Radiotherapy+ Chemotherapy | Yes | Lung, lymph nodes, pleura, mediastinum, liver, diaphragmatic muscle, bone | NA | >6 |
| Kim et al. (2017) | 10 | Male | Left frontoparietal lobe | Surgery | Yes | Scalp, temporalis muscle, lung, liver, buttock, bone, mediastinal lymph nodes | Resection+ Chemotherapy | 9 |
| Umbach et al. (2020) | 17 | Female | Left frontal lobe | Surgery | Yes | Right parotid gland, bilateral cervical lymph nodes | None | >7 |
| Joris et al. (2022) | 12 | Male | Posterior fossa | Surgery | NA | Lumbosacral bone | Resection | NA |
| Zhou et al. (2022/Present study) | 9 | Female | Posterior fossa | Surgery+ Radiotherapy | Yes | Sphenoid sinus, bone and lung | Resection+ Chemotherapy | >3 |
